# Pilot study examining the effect of rurality on engagement and abstinence for adult users of a text-message cessation intervention

**DOI:** 10.1186/s12889-025-25284-6

**Published:** 2025-11-19

**Authors:** James W. Kinchen, Melissa A. Little, Lee M. Ritterband, Kara P. Wiseman

**Affiliations:** 1 Department of Public Health Sciences, The University of Virginia School of Medicine, Charlottesville, VA USA; 2 Center for Behavioral Health & Technology, Department of Psychiatry and Neurobehavioral Sciences, The University of Virginia School of Medicine, Charlottesville, VA USA

**Keywords:** Cessation, Rurality, MHealth, Text messaging, Tobacco use

## Abstract

**Background:**

Mobile health (mHealth) interventions have the potential to expand the reach of smoking cessation interventions. However, it is unknown if people using the same cessation mHealth intervention but living in different places have similar program engagement, satisfaction, and outcomes. The objective of this study was to explore the potential need to enhance existing interventions for rural populations by examining program engagement, satisfaction, and outcomes by rurality for the publicly available cessation text messaging intervention, SmokefreeTXT.

**Methods:**

Participants were adults interested in quitting smoking from Virginia (*N* = 49), with recruitment stratified by county-level rurality (*n* = 23 rural, *n* = 26 non-rural). Participants completed a 7-week version of SmokefreeTXT and completed assessments at baseline, immediately post-intervention, and three- and six-months from enrollment. Descriptive statistics compared program engagement, satisfaction, and seven-day point prevalence abstinence at all time points by rurality. Multivariable logistic regression models assessed the association between rurality and cessation at all time points.

**Results:**

Overall, seven-day abstinence rates were 32.6%, 28.6%, and 36.7%, and among participants from rural counties rates were 39.1%, 30.4%, and 43.5% at post-intervention, three-, and six-months, respectively. Participants from rural counties had higher use of real-time support for mood and cravings and reported this support to be more useful than participants from non-rural counties (Ps < 0.05).

**Conclusions:**

mHealth cessation interventions may work well for people living in rural counties, potentially providing good support for adults attempting to quit smoking but lacking easy access to traditional cessation support services. Modifications to enhance support for craving and mood may be particularly relevant for people living in rural areas.

**Trial registration:**

ClinicialTrials.gov: NCT05055778, Registration date: 9/14/2021.

**Supplementary Information:**

The online version contains supplementary material available at 10.1186/s12889-025-25284-6.

## Background

Smoking continues to be a leading cause of preventable morbidity and mortality in the United States (U.S.), leading to more than 480,000 deaths annually [1]. Despite a general decline in tobacco use in the U.S. over the last 70 years, disparities have persisted, with tobacco use declining in rural areas at a slower rate than non-rural areas [2]. Currently, it is estimated that nationally, smoking rates in rural areas are approximately 19%, compared to approximately 14% in non-rural areas [3]. At the state level, differences in smoking rates between rural and non-rural areas can be even more pronounced. For example, in Virginia, the late-2024 estimated smoking rate in one rural health district (Lenowisco) is 26.4%, compared to 3.4% in a non-rural health district (Arlington) [4]. Trends in rural tobacco use can be attributed to socioeconomic factors [5, 6] and social normalization of tobacco [7–9]. Further, areas where tobacco agriculture is a main industry have additional exposure risk and use is more socially acceptable [10, 11]. Additionally, previous research has documented that adults living in medically underserved areas, such as rural areas, have higher smoking rates [12] and are less likely to have access to cessation programs [13–15]. 

Despite evidence of the benefits of smoking cessation and interest in quitting smoking among those who currently smoke, fewer than 10% of adults who smoke make a successful quit attempt annually [16]. The low success rate could indicate a need for updated and increased support for those who are interested in quitting [16, 17]. There is evidence that socially or culturally adapted cessation programs might have increased engagement and success in supporting long-term abstinence [18, 19]. 

As technology (e.g., broadband, cellular service) becomes more available in rural areas and cell phone ownership increases, mHealth interventions are becoming a key method to making cessation programming more available [20–23]. In particular, text messaging cessation programs are one of the most cost-efficient forms of cessation support and are a well-supported method by which smoking cessation interventions can be provided to large numbers of people [24–26]. Text-message based cessation programs are already available, free of charge, to adults throughout the country through state quit lines and the National Cancer Institute (NCI) Smokefree.gov Initiative program, SmokefreeTXT.

While mHealth interventions can address barriers related to access to evidence-based programs, the impact of rurality on program engagement, satisfaction, or cessation outcomes are unknown, and could differ by rurality. For example, specific messages within SmokefreeTXT may not resonate with adults living in rural areas or gaps in cellular service coverage may impact program delivery. Thus, it is necessary to understand how well currently available text-message based interventions work for rural populations to determine if there is a need to develop new or enhance existing interventions by tailoring to the needs of rural populations. The aims of this pilot study were to evaluate engagement, satisfaction, and cessation outcomes by rurality among adults using SmokefreeTXT.

## Methods

### Study population and procedure

Participants included 49 adults currently smoking and living in Virginia. Recruitment occurred from November 2021 to August 2023 using local community partners from across Virginia (e.g., Blue Ridge Health District, Virginia Premier) and Facebook advertisements. Interested potential participants completed an online interest form to determine eligibility and consent to participate. Eligibility criteria included being at least 18 years of age, currently smoking, residing in Virginia, speaking English, and owning a cell phone. Anyone currently attempting to quit, and women who were currently pregnant or who anticipated becoming pregnant within 6 months, were excluded. Any potential participant whose identity could not be verified was also excluded [27]. The zip code of interested participants was used to determine county-level rurality using Rural-Urban Commuting Area Codes (RUCA) codes [28], with the goal of recruiting an equal number of participants from rural (RUCA code 4+) and non-rural (RUCA code 1–3, grouping suburban and urban together) counties across Virginia. After completing the study screener and informed consent, and completing identity verification [27], participants who met eligibility criteria were asked to complete a baseline assessment. After completing the baseline assessment, participants were given instructions on how to enroll in SmokefreeTXT. Participants were considered enrolled in the study once study staff were able to confirm that a participant had completed the baseline assessment and enrolled in SmokefreeTXT. Of 120 completed interest forms, 69 adults were deemed eligible, with 49 fully enrolled (Supplementary Figure). As a pilot study designed to establish initial cessation rates and test program acceptability, power calculations were not completed and thus this study is assumed to not be powered to identify statistical differences in outcomes by rurality. All study procedures took place remotely using participants’ own phone for intervention delivery. This study was approved by the Social and Behavioral Sciences IRB at the University of Virginia (SBS IRB# 3928) in accordance with 45 CFR 46 (2018 Code of United States Federal Regulations - Public Welfare: Protection of Human Subjects), which is in compliance with the Helsinki Declaration and Belmont Report.

### SmokefreeTXT

SmokefreeTXT is a free, publicly available text-messaging smoking cessation intervention hosted by the NCI Smokefree.gov Initiative [29, 30]. SmokefreeTXT uses automated text messages with two-way interaction to support cessation [31, 32]. During the intervention, participants receive 3–5 motivational and informational text messages daily for 6–8 weeks, which includes two weeks of preparation messages and 6 weeks of cessation messages. The intervention is fully automated with messages timed around the quit date. Automated support and accountability are provided at any time with the use of keywords for cravings, stress, and mood [33]. Users are able to change their quit date to restart SmokefreeTXT at any time. For this study, a slightly standardized version of the program was used in which the setting of the participants’ initial quit date was made one week from enrollment rather than allowing the user to select their own initial quit date within two weeks of enrollment as is done in the publicly available version. This allowed for standardization of initial quit dates across all study participants. Participants retained the ability to reset their quit date at any time. All other messages and functionality were identical between the publicly available and standardized versions. Use of a standardized, in-house version also allowed for the study team to monitor participant process through the program and to have access to study participant data.

### Data and coding

#### Assessment data

Participants were asked to complete four online assessments: baseline, post-intervention (7-weeks after enrollment), three-, and six-months after enrollment. Participants were sent emails with links to each assessment and study staff sent text message reminders to increase response rates. The baseline assessment included items to measure tobacco use history (e.g., age at first cigarette, frequency of smoking, other tobacco product use), nicotine dependence [34], readiness and motivation to quit, confidence in quitting, previous quit attempts, cell phone and technology use, cell service quality, neighborhood social cohesion [35], unmet needs [36, 37], depression and anxiety [38], alcohol use, and demographic characteristics (age, gender, race, education, employment status, marital status, and sexual orientation). The post-intervention, three-, and six-month assessments measured the primary outcome of interest: seven-day point prevalence smoking abstinence (responses to the question: “*Have you smoked a cigarette (even a puff) in the past 7 days?”*, with responses of ‘yes’ and ‘no’). To provide the most conservative estimates of abstinence, missing responses for each assessment were coded as continuing to smoke. Additionally, follow-up assessments measured: non-cigarette tobacco use, nicotine dependence [34], reasons for continued smoking (among those who reported continued smoking), motivations and confidence in quitting, cessation techniques used during the previous assessment period, alcohol use, depression and anxiety [38], and satisfaction with and usefulness of SmokefreeTXT. Satisfaction was measured by asking participants *“So far*,* how much has the program helped you: stay motivated about quitting smoking*,* resist cravings*,* helped you feel supported*,* and quit smoking?”.* Satisfaction had response options of “Not at all”, “A little bit”, “Some”, “A good deal”, and “A lot”. Usefulness was measured by asking *“How useful were the following: messages about cravings*,* messages about staying motivated*,* messages about reasons to quit*,* ability to reset your quit date*,* real-time support for cravings*,* real-time support for mood*,* real-time support for slips*,* questions asking about my mood*,* questions asking about my cravings*,* questions asking about my smoking status*,* and links to get more information?”.* Usefulness had response options of “Extremely Useful”, “Somewhat Useful”, “Not at All Useful”, and “Don’t Remember”. Satisfaction with message frequency was assessed as “Not Enough Messages”, “About the Right Number of Messages”, and “Too Many Messages”. Response rates to the follow-up assessments were 83.7% at post-intervention (rural: 78.3%, non-rural: 84.6), 77.6% at three-months (rural: 73.9%, non-rural: 88.5%), and 77.6% at six-months (rural: 78.3%, non-rural: 76.9%).

#### Program data

Every message sent by participants to the standardized version of SmokefreeTXT was date and timed stamped, allowing for investigation of intervention engagement. Engagement was operationalized as responding to within-SmokefreeTXT questions about readiness to quit on the quit day, craving, mood, and smoking status (responded or not to each question) and use of the real-time support keywords, “CRAVE”, “STRESS”, and “MOOD” (number of uses of each keyword, respectively). Use of the keyword, “NEW”, which allowed participants to re-set or change their quit date, was used to define the number of cycles of SmokefreeTXT each participant completed. A cycle was defined as the set of program data associated with a specific quit date from the time that quit date was implemented until the time that the keyword “NEW” was used or until the intervention was completed. For participants with multiple cycles, the cycle with the longest time between resetting and completing the intervention was used for analyses.

### Statistical analyses

The primary outcome of interest was self-reported 7-day smoking abstinence measured at each follow-up time point. Secondary outcomes of interest included self-reported satisfaction and usefulness, and engagement with SmokefreeTXT. Descriptive statistics were used to characterize the sample, describe cessation outcomes, and estimate satisfaction and engagement overall and by rurality using t-tests, Chi-Squared tests, and Fisher’s exact tests as appropriate; however, we were not powered to detect differences by rurality. Logistic regression models estimated the association between rurality (rural vs. non-rural county) and abstinence (outcome reference = continued smoking) at each follow-up time point. First, a crude model determined the association between rurality and abstinence for each follow-up time point. Then, baseline characteristics known to be associated with cessation based on the existing literature (multiple previous quit attempts, binge drinking, time to first cigarette, confidence to quit, and unmet needs) were considered for inclusion in a final multivariable model using the change in estimate method [39]. The final models controlled for binge drinking, time to first cigarette, confidence to quit (all time points), and unmet needs (three-month follow-up only). Sensitivity analyses repeated the final multivariable logistic regression models only among assessment responders and explored using a repeated measures model. Listwise deletion was used for missing data in all analyses. All analyses were conducted using SAS 9.4 (SAS Institute Inc., Cary, NC).

## Results

Just over half of participants (53.1%) were from non-rural counties in Virginia and 46.9% were from rural counties. Participants were primarily female (83.7%) and non-Hispanic white (73.5%, Table 1). The mean age at enrollment was 46.3 years (SD = 11.9 years). The majority of participants (91.8%) had at least a high school degree and over half (55.1%) reported being employed. Nearly all participants (98%) reported good or excellent cell service at their home (rural: 95.6%; non-rural: 100%, Χ^2^ = 1.154, *p* = 0.47) and most (75.5%) reported good or excellent cell service at work or school (rural: 69.6%; non-rural: 80.8%, Χ^2^ = 2.501, *p* = 0.32). At baseline, 10.2% of participants smoked some days and 89.8% reported smoking every day. Almost one quarter of participants (24.5%) had very high nicotine dependence (rural: 39.1%, non-rural: 11.5%, Χ^2^ = 5.399, *p* = 0.25) with the majority (75.5%) reporting smoking 11 or more cigarettes per day (rural: 72.6%, non-rural: 69.2%, Χ^2^ = 1.191, *p* = 0.55) and almost half (44.9%) reporting smoking their first cigarette within five minutes of waking (rural: 60.9%, non-rural: 30.8%, Χ^2^ = 5.139, *p* = 0.08). Measures of nicotine dependence were descriptively higher for participants from rural counties, but comparisons did not reach statistical significance. Multiple tobacco product use was common, with 46.9% of participants also using non-cigarette tobacco products (rural: 43.5%, non-rural: 50.0%, Χ^2^ = 0.208, *p* = 0.78).

**Table 1:** Sample demographic and tobacco use characteristics

				Total *N* = 49		Rural *n* = 23 (46.9%)		Non-Rural *n* = 26 (53.1%)			
				Mean (SD)		Mean (SD)		Mean (SD)		t-value	*p*-value
Age at Enrollment, years				46.3 (11.9)		46.2 (10.4)		46.3 (13.3)		0.01	0.99
Unmet Needs Score				2.65 (2.18)		3.09 (2.11)		2.27 (2.20)		-1.32	0.19
Confidence to Quit in Next 6 Months				2.12 (0.90)		1.87 (0.97)		2.35 (0.80)		1.89	0.07
				**n/N**	**(%)**	**n/N**	**(%)**	**n/N**	**(%)**	**Χ** ^**2**^	***p*** **-value**
Race/Ethnicity										1.074	0.65
NH White				36/49	(73.5)	16/23	(69.6)	20/26	(76.9)		
NH Black				7/49	(14.3)	3/23	(13.0)	4/26	(15.4)		
Other^a^				6/49	(12.2)	4/23	(17.4)	2/26	(7.7)		
Gender^b^										0.509	0.83
Male				6/49	(12.2)	2/23	(8.7)	4/26	(15.4)		
Female				41/49	(83.7)	20/23	(87.0)	21/26	(80.8)		
Sexual Orientation										0.508	0.67
Heterosexual				43/49	(87.8)	21/23	(91.3)	22/26	(84.6)		
Gay, Lesbian, or Bisexual				6/49	(12.2)	2/23	(8.7)	4/26	(15.4)		
Occupational Status										0.240	1.00
Employed				27/49	(55.1)	13/23	(56.5)	14/26	(53.8)		
Unemployed				3/49	(6.1)	1/23	(4.4)	2/26	(7.7)		
Other^c^				19/49	(38.8)	9/23	(39.1)	10/26	(38.5)		
Education Level										1.407	0.76
Less than High School				4/49	(8.2)	1/23	(4.3)	3/26	(11.5)		
High School/GED				13/49	(26.5)	6/23	(26.1)	7/26	(26.9)		
Some College				18/49	(36.7)	8/23	(34.8)	10/26	(38.5)		
College Graduate or More				14/49	(28.6)	8/23	(34.8)	6/26	(23.1)		
Marital Status^d^										1.422	0.35
Single^e^				26/43	(60.5)	14/20	(70.0)	12/23	(52.2)		
Married/Partnered				17/43	(39.5)	6/20	(30.0)	11/23	(47.8)		
Daily Smoking Status										0.108	1.00
Every Day				44/49	(89.8)	21/23	(91.3)	23/26	(88.5)		
Some Days				5/49	(10.2)	2/23	(8.7)	3/26	(11.5)		
Time to First Cigarette after Waking										5.139	0.08
< 5 min				22/49	(44.9)	14/23	(60.9)	8/26	(30.8)		
6–30 min				18/49	(36.7)	5/23	(21.7)	13/26	(50.0)		
31 min or Longer				9/49	(18.4)	4/23	(17.4)	5/26	(19.2)		
Number of Cigarettes Per Day										1.191	0.55
< 10				12/49	(24.5)	4/23	(17.4)	8/26	(30.8)		
11–20				27/49	(55.1)	14/23	(60.9)	13/26	(50.0)		
21 or More				10/49	(20.4)	5/23	(21.7)	5/26	(19.2)		
Nicotine Dependence^f^										5.399	0.25
Very Low				3/49	(6.1)	1/23	(4.4)	2/26	(7.7)		
Low				7/49	(14.3)	2/23	(8.7)	5/26	(19.2)		
Medium				7/49	(14.3)	3/23	(13.0)	4/26	(15.4)		
High				20/49	(40.8)	8/23	(34.8)	12/26	(46.2)		
Very High				12/49	(24.5)	9/23	(39.1)	3/26	(11.5)		
Menthol										0.525	0.57
Menthol				24/49	(49.0)	10/23	(43.5)	14/26	(53.9)		
Non-Menthol				25/49	(51.0)	13/23	(56.5)	12/26	(46.2)		
Use Non-Cigarette Tobacco Products										0.208	0.78
Yes				23/49	(46.9)	10/23	(43.5)	13/26	(50.0)		
No				26/49	(53.1)	13/23	(56.5)	13/26	(50.0)		
Cell Phone Service Quality at Home^g^										1.154	0.47
Poor/Fair				1/49	(2.0)	1/23	(4.4)	0/26	(0.0)		
Good/Excellent				48/49	(98.0)	22/23	(95.6)	26/26	(100.0)		
Cell Phone Service Quality at Work or School										2.501	0.32
Poor/Fair				2/49	(4.1)	2/23	(8.7)	0/26	(0.0)		
Good/Excellent				37/49	(75.5)	16/23	(69.6)	21/26	(80.8)		
Does Not Apply				10/49	(20.4)	5/23	(21.7)	5/26	(19.2)		
Cell Phone Service Quality in Town^d,g^										0.001	1.00
Poor/Fair				2/47	(4.3)	1/23	(4.4)	1/24	(4.2)		
Good/Excellent				45/47	(95.7)	22/23	(95.6)	23/24	(95.8)		

^a^Includes Non-Hispanic Asian, Hispanic White, Hispanic Black, Hispanic Asian, Non-Hispanic Other, and Hispanic Other

^b^Does not equal 100% due to censoring of small cell sizes of additional categories of gender-identity that were collected

^c^Includes Homemaker, Student, Retired, Disabled, or some other status

^d^Missing data treated as missing.

^e^Includes Single Never Married, Divorced, Widowed, and Separated

^f^Based on the Fagerstrom Nicotine Dependence Score; Score of: 0–2 = Very Low, 3–4 = Low, 5 = Medium, 6–7 = High, 8–11 = Very High

^g^No respondents to response option “Does No Apply” for this item.

Seven-day abstinence was 32.6%, 28.6%, and 36.7% at post-intervention, three-months, and six-months, respectively (Table 2). Cessation rates for participants from rural counties were 39.1%, 30.4%, and 43.5% for post-intervention, three-months, and six-months, respectively versus 26.9%, 26.9%, and 30.8%, for participants from non-rural counties. Rurality was not associated with cessation in final logistic regression models (Table 2 and Supplemental Table 1). Patterns were consistent when only assessment responders were included as a sensitivity analysis (data not shown).

**Table 2:** Smoking cessation at each assessment timepoint (*N* = 49)

		Smoked^1^		Did Not Smoke			
		*n*/*N* (%)	95%CI	*n*/*N* (%)	95%CI	*p*-value^2^	OR(95% CI)^3^
End of intervention							
Total		33/49 (67.4)	(53.74, 80.96)	16/49 (32.6)	(19.04, 46.26)		
Rural		14/23 (60.9)	(40.20, 81.54)	9/23 (39.1)	(18.46, 59.80)	0.36	4.13 (0.90-18.88)
Non-Rural		19/26 (73.1)	(55.41, 90.75)	7/26 (26.9)	(9.25, 44.59)		ref
3 Months							
Total		35/49 (71.4)	(58.32, 84.54)	14/49 (28.6)	(15.46, 41.68)		
Rural		16/23 (69.6)	(50.07, 89.06)	7/23 (30.4)	(10.94, 49.93)	0.79	2.05 (0.45–9.39)
Non-Rural		19/26 (73.1)	(55.41, 90.75)	7/26 (26.9)	(9.25, 44.59)		ref
6 months							
Total		31/49 (63.3)	(49.27, 77.26)	18/49 (36.7)	(22.74, 50.73)		
Rural		13/23 (56.5)	(35.52, 77.52)	10/23 (43.5)	(22.48, 64.48)	0.36	3.16 (0.74–13.48)
Non-Rural		18/26 (69.2)	(50.84, 87.62)	8/26 (30.8)	(12.38, 49.16)		ref

^a^Non-responders coded as continuing to smoke

^b^Adjusted for Binge Drinking (all time points), Time to First Cigarette (all time points), Confidence to Quit in the Next 6 Months (all time points), and Unmet Needs (3-months only)

^2^*p*-value from Rao-Scott Chi-Square Test

**Fig. 1:**
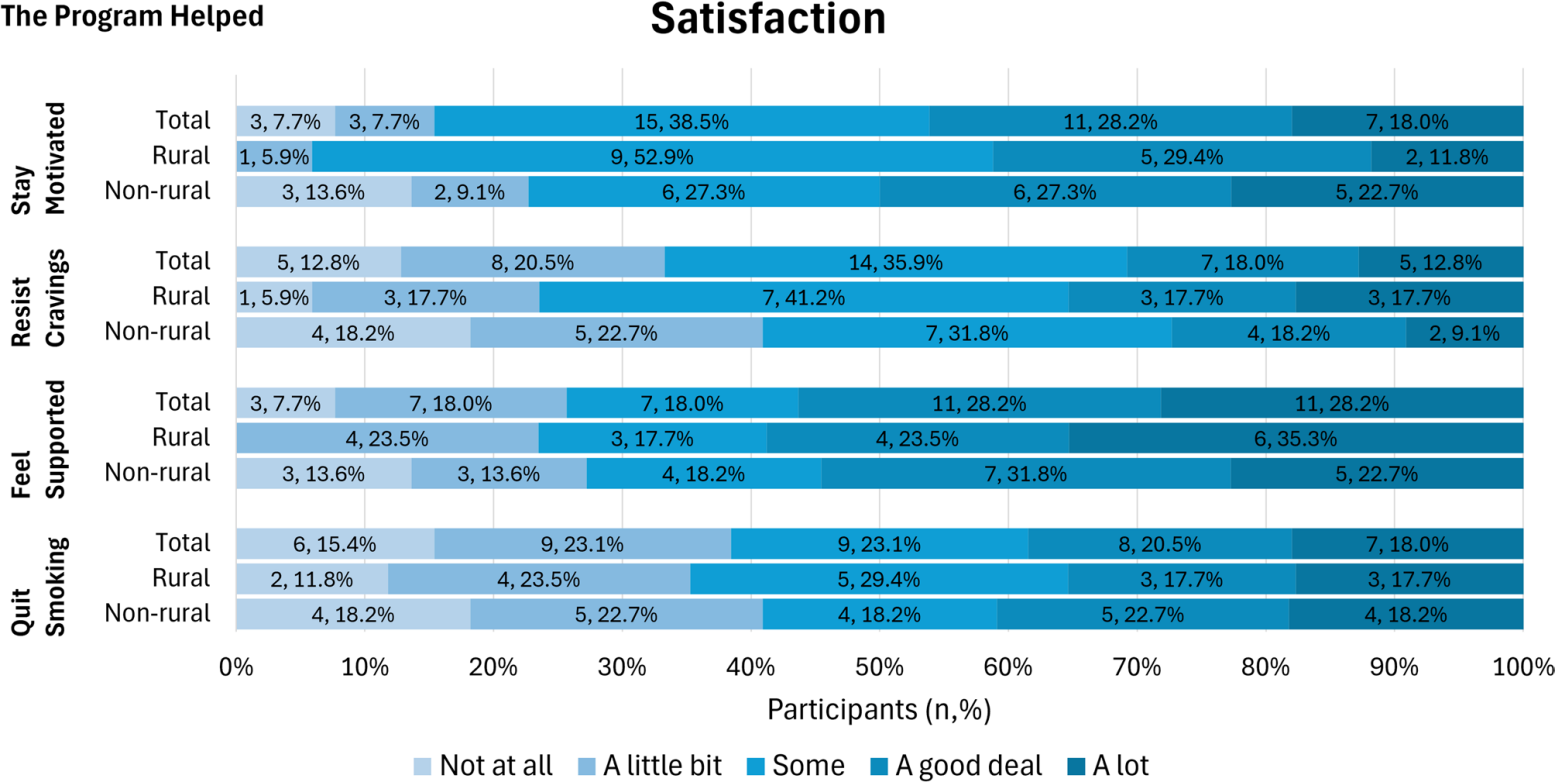
Self-reported satisfaction with SmokefreeTXT at the end of intervention assessment. Responses reported are from *n* = 39 (*n* = 17 rural, *n* = 22 non-rural) participants who responded to these items on the post-intervention survey

**Fig. 2:**
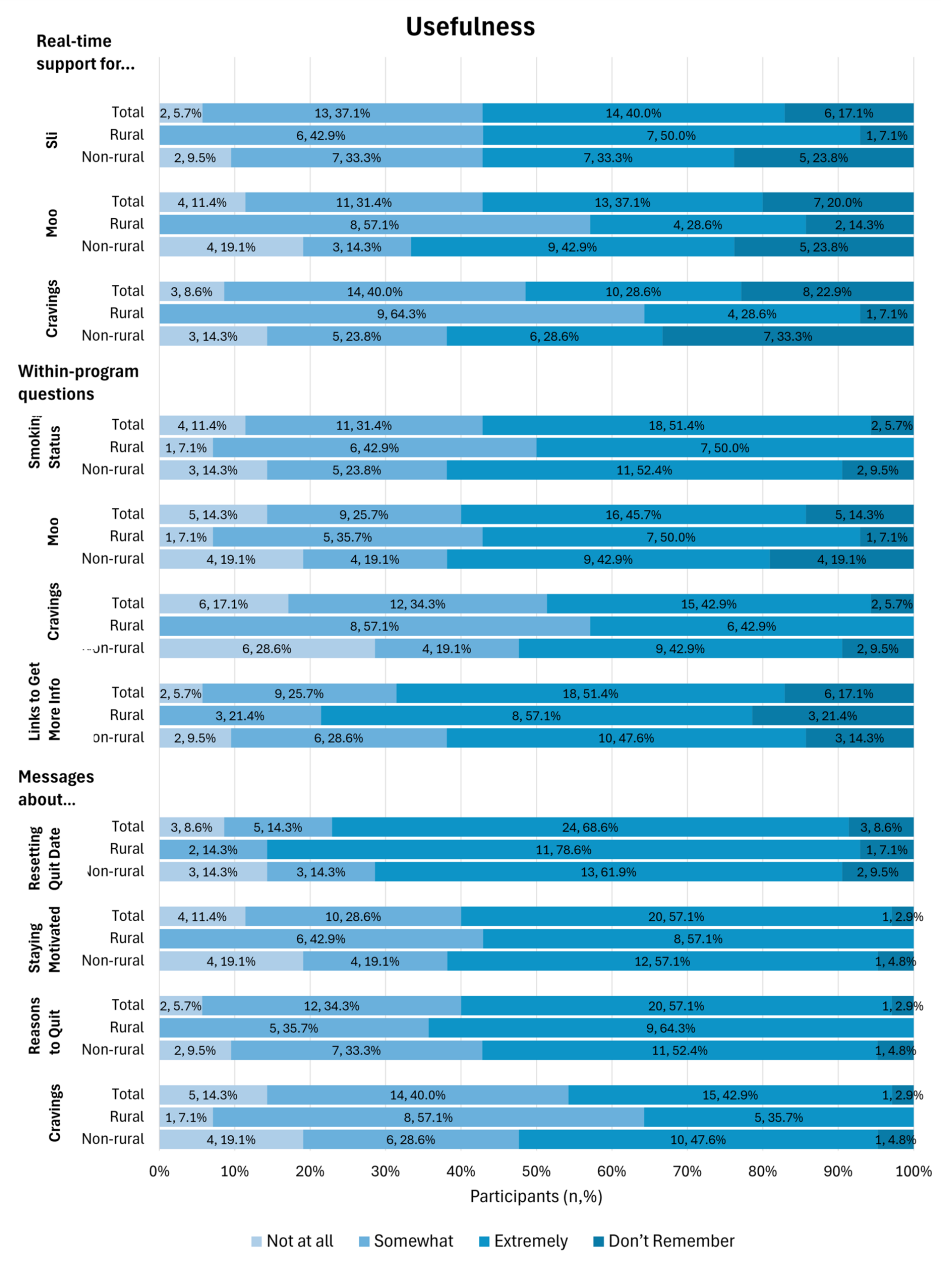
Self-reported usefulness of SmokefreeTXT at the end of intervention assessment. Responses reported are from *n* = 35 (*n* = 14 rural, *n* = 21 non-rural) participants who responded to these items on the post-intervention survey. “Real-Time Support” refers to messages triggered by a user by texting key words

Overall, participants were satisfied with SmokefreeTXT and found it to be useful in their quit attempts. Participants had the highest level of satisfaction with feeling supported by SmokefreeTXT, with 56.4% reporting that the program helped them feel supported (Fig. 1). The three intervention features reported most often as being extremely useful were the ability to reset a quit date (68.9%), messages about reasons to quit (57.1%), and messages about staying motivated (57.1%, Fig. 2). Participants from rural counties reported automated real-time support for mood and cravings to be more useful compared to participants from non-rural counties. For cravings, participants from rural counties found this support to be somewhat (64.3%) or extremely (28.6%) useful compared to 23.8% and 28.6%, somewhat or extremely useful, respectively, for participants from non-rural counties (*p* = 0.049). For mood, participants from rural counties found this support somewhat (57.1%) or extremely (28.6%) useful compared to 14.3% and 42.9%, somewhat or extremely useful, respectively, for participants from non-rural counties (*p* = 0.047). Participants from rural counties found questions assessing craving levels to be somewhat (57.1%) or extremely (42.9%) useful compared to 19.1% and 42.9% somewhat or extremely useful, respectively, for participants from non-rural counties (*p* = 0.025).

**Fig. 3:**
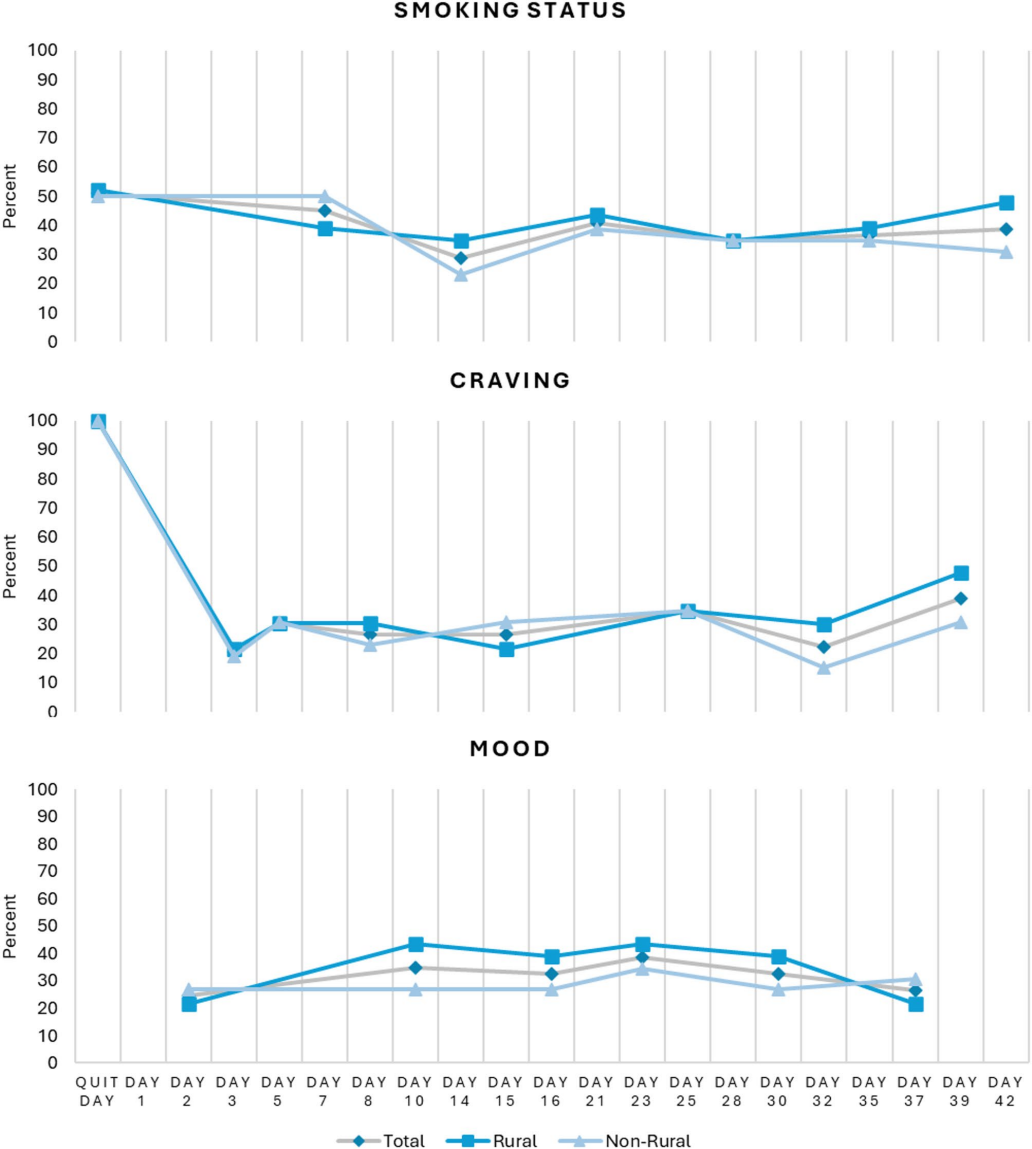
SmokefreeTXT engagement, overall and by rurality. Response rates to within program questions on smoking status, craving level and mood by rurality. Response rates are calculated based on the full study sample of *N* = 49 (*n* = 23 rural, *n* = 26 non-rural) during each participant’s longest cycle of SmokefreeTXT. Participants that opted out are categorized as non-responders for questions sent after their opt-out date

**Fig. 4:**
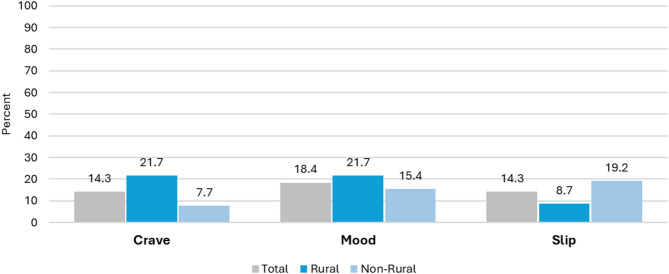
SmokefreeTXT Real-Time Support Utilization. Percent of participants that used SmokefreeTXT keywords (crave, mood, and slip) for real-time support by rurality. “Real-Time Support” refers to messages triggered by a user by texting key words. Calculated based on the full study sample of *N* = 49 (*n* = 23 rural, *n* = 26 non-rural) during their longest cycle of SmokefreeTXT

Over half (55.1%) of participants reset their quit date at least once, resulting in multiple cycles through SmokefreeTXT. Among those with multiple cycles, 33% (*n* = 16) had highest engagement during their second attempt, 12% (*n* = 6) during their third attempt, 6% (*n* = 3) during their fourth attempt, and 4% (*n* = 2) during their sixth attempt (data not shown). With the exception of craving status on the quit day (100% response rate), response rates to the within-SmokefreeTXT questions about cravings and mood were low, ranging from 20.4% to 38.8% overall, 21.4% to 47.8% among rural, and 15.4% to 34.6% among non-rural (Fig. 3). Less than 55% of participants responded to the weekly question asking for current smoking status sent within SmokefreeTXT (rural: 42%, non-rural: 37%). The average overall response rate was 21%, or each participant answering, on average, 1.5 out of the 7 smoking status questions sent during the intervention (average response rate rural: 16.2%, non-rural: 25.3%, *p* = 0.243). Response rates were highest on Day 42 for participants from rural counties (47.8%) and Day 7 for participants from non-rural counties (50.0%). The keywords CRAVE, MOOD, and SLIP were used by 14.3%, 18.4%, and 14.3% of participants, respectively (Fig. 4). Among rural participants, 21.7%, 21.7%, and 8.7% used the keywords CRAVE, MOOD, and SLIP, respectively; among non-rural participants, 7.7%, 15.4%, and 19.2%, respectively. Patterns of keyword use differed by rurality with participants from non-rural counties using the slip keyword most frequently, compared to participants from rural counties who had highest and equal use of the CRAVE and MOOD keywords.

## Discussion

mHealth interventions have considerable potential to extend access of cessation support to rural populations, which experience higher tobacco use rates yet reduced access to clinic-based cessation support services. This study aimed to evaluate engagement, satisfaction, and cessation outcomes from use of the publicly available intervention, SmokefreeTXT, by rurality. We found that SmokefreeTXT was able to support adults in a quit attempt regardless of where they live. However, there were notable differences between participants living in rural versus non-rural counties in engagement with SmokefreeTXT and self-reported usability. Specifically, participants from rural counties reported higher usefulness for real-time support for cravings and mood compared to participants from non-rural counties. They also used these features more often, which points to a potential opportunity to identify novels methods to support craving and mood to increase program usefulness and effectiveness for this population.

With limited access to traditional in-person cessation support services, modern mHealth text-message based interventions may provide an important, scalable resource to support cessation for people living in rural areas. While other studies have examined use of SmokefreeTXT by race or by sexual orientation, to our knowledge, this is the first study to compare a text-message based cessation program by rural and non-rural counties [29, 40]. Studies that have explored cessation interventions by rurality have focused primarily on in-person support services and nicotine replacement therapy use. One study by Northridge et al. (2008) found that among users of group cessation classes, fewer participants from rural areas in West Virginia successfully quit compared to their peers from non-rural areas (51.4% vs. 60.6%) [41]. Another study by Little et al. (2024) explored feasibility of delivering nicotine replacement therapy (NRT) in rural areas through local community pharmacies, finding that participants from rural areas utilized NRT in high amounts and that these participants had a 31.8% 7-day abstinence rate at 3-months [42], similar to our study. While the current study was not powered to detect differences between rural and non-rural participants with respect to cessation related outcomes, rural participants had descriptively higher cessation rates across the three time points compared to non-rural participants. Given the limited knowledge about the impact of rurality on cessation intervention outcomes, additional research, with larger, more generalizable samples, is needed to explore the effects of rurality on mHealth cessation interventions across the United States.

We found that the ability to receive real-time supportive messages during periods of high cravings was of particular interest to participants from rural counties. It is possible that this interest was due to the high level of nicotine dependence observed among rural participants in the study population. Previous studies have also documented moderate to high levels of nicotine dependence in rural populations [43–45]. Our sample was not large enough to consider the intersection of nicotine dependence and rurality with cessation or SmokefreeTXT utilization and satisfaction; therefore, future research is needed to examine the influence of nicotine dependence on cessation by rurality.

Engagement in mHealth interventions is considered an important factor yet is not yet fully understood. Several different operationalizations of engagement were considered in this study, with each one representing a unique facet of the underlying theory of engagement [46]. Specifically, we considered use of on-demand support to represent dose, as more use of keywords would increase the total dose of intervention received. In contrast, opting-out was also conceptualized to represent dose but in the opposite direction, with the decision to opt-out reducing overall intervention dose. Response to within program questions was conceptualized as representing affect as feelings about the intervention itself or a participant’s success in quitting could influence interest in responding. Lastly, re-setting a quit date after a smoking lapse was conceptualized as representing interest in both the intervention and the target behavior. When considering response rates to within-SmokefreeTXT cessation questions, engagement in SmokefreeTXT was generally low, ranging from 28.6% to 51.0% overall. However, these rates are higher than what has been reported in previous explorations of engagement with SmokefreeTXT when implemented as a publicly available program [29, 30]. For example, response rates ranged from 7.4% to 19.9% and 14.3% to 31.3% among Black and White adults, respectively, who had signed up for SmokefreeTXT [29]. It is possible that our participants were more engaged due to being enrolled in a research study. Alternatively, there was high utilization of the ability for participants to re-set their quit date, showing sustained interest in using SmokefreeTXT and quitting smoking. High use of this feature in particular, might mean that a more adaptive intervention to specifically support people who make multiple quit attempts is needed. Future research should explore how to increase overall engagement to support the creation of adaptive interventions within text-messaging and seek to identify optimized engagement levels needed to support behavior change. While it is possible that low engagement could be due to issues receiving the messages, cellular service was not a problem in our study. In fact, participants indicated good to excellent cell service at home, on their property, and in town regardless of rurality, which was also evidenced by the very low number of carrier errors or undeliverable messages that occurred during this study (data not shown). With major improvements to cellular infrastructure over the last few years, and more than 90% of Americans owning cell phones [47, 48], the historical belief that people living in rural areas are not able to access mHealth interventions may need to be revisited. mHealth interventions have great potential for efficient scaling and could help close the access gap for clinical cessation support [24–26], ultimately reducing smoking rates and tobacco-related morbidity and mortality.

Adults who smoke and live in rural areas have documented differences in tobacco use patterns, lifestyles, cultures, and beliefs compared to people living in non-rural areas. However, to our knowledge, only one text-messaging based cessation support programs specifically designed for smokers living in rural areas has been developed [24]. This program implemented a four-week gradual smoking reduction intervention, however, it has not yet been tested for long-term cessation outcomes. Even with the promising initial cessation rates found in the current study, small increases in the effectiveness of SmokefreeTXT for rural populations through creation of a tailored program could result in clinically meaningful outcomes. Additionally, given the scale of SmokefreeTXT utilization already, small increases in efficacy can also result in population health impact. There is some evidence that tailoring enhances intervention effectiveness for rural populations. For example, for veterans living in rural areas, tailored counseling for cessation based on rurality has been observed to produce higher odds of 3-month cessation compared to referral to a state quit line (OR = 1.83; 95% CI 1.16–2.88) [43]. There is evidence that it is possible to culturally tailor text-message based interventions. For example, messages culturally tailored to the beliefs of rural American Indian tribes have been found to be better received by tribe members [18]. Similarly, tailoring SmokefreeTXT for sexual and gender minority groups has been shown to increase engagement and satisfaction [40]. The identified differences in self-reported usefulness and utilization found in this study point to the potential benefit of tailoring for rurality to increase engagement and cessation outcomes for text-messaging based cessation interventions. Future studies should explore the impact of tailored support based on rurality to SmokefreeTXT on engagement and cessation rates.

Our study was not without its limitations. First, this pilot study implemented a standardized version of SmokefreeTXT in Virginia with a modest sample size of 49. Although, our goal of recruiting participants across the state and from rural and non-rural counties was achieved, the small sample limited our ability to compare the effectiveness of SmokefreeTXT between rural and non-rural participants with sufficient statistical power. Our sample was also predominately female; thus, these results may not be generalizable to men who currently smoke. Additionally, abstinence was collected via self-report and biochemical verification was not used, potentially introducing self-reporting bias. Our sample reported high levels of cell service coverage and cellular device access, which might limit generalizability to other areas with lower levels of cell service coverage and device access. Despite these limitations, we were able to successfully identify SmokefreeTXT as a viable cessation tool for people who smoke and live in rural areas and identify new areas of investigation to enhance an existing and highly utilized publicly available cessation intervention.

## Conclusion

mHealth cessation interventions work well across rurality, with the potential to provide particularly good support to adults attempting to quit smoking but lacking easy access to traditional cessation support services, including for those with high levels of nicotine addiction. Participants from rural counties reported greater levels of perceived usefulness of the automated support offered by SmokefreeTXT for cravings and mood support, indicating potential areas for intervention innovation to support cessation. In particular, tailoring SmokefreeTXT to target participants with high levels of nicotine addiction through addition of supplemental craving and nicotine withdrawal support, or mindfulness exercises to improve mood could be beneficial to this population. With continued high prevalence of smoking in rural areas, mHealth interventions may be a useful way to provide effective cessation resources to adults in need of cessation support.

## Supplementary Information

Supplementary Material 1.

Supplementary Material 2.

## Data Availability

The datasets used and/or analyzed during the current study are available from the corresponding author on reasonable request.

## Abbreviations

U.S.: United States
NCI: National Cancer Institute
mHealth: Mobile Health
RUCA: Rural–Urban Commuting Area
IRB: Institutional Review Board
NRT: Nicotine Replacement Therapy
