# Supplementary material for: Pilot study examining the effect of rurality on engagement and abstinence for adult users of a text-message cessation intervention
Source: BMC Public Health. 2025 Nov 19;25:4061. doi: 10.1186/s12889-025-25284-6 (PMC12629046; doi:10.1186/s12889-025-25284-6)
Supplement: Supplementary file 1 — Supplementary Material 1. [file 12889_2025_25284_MOESM1_ESM.docx]

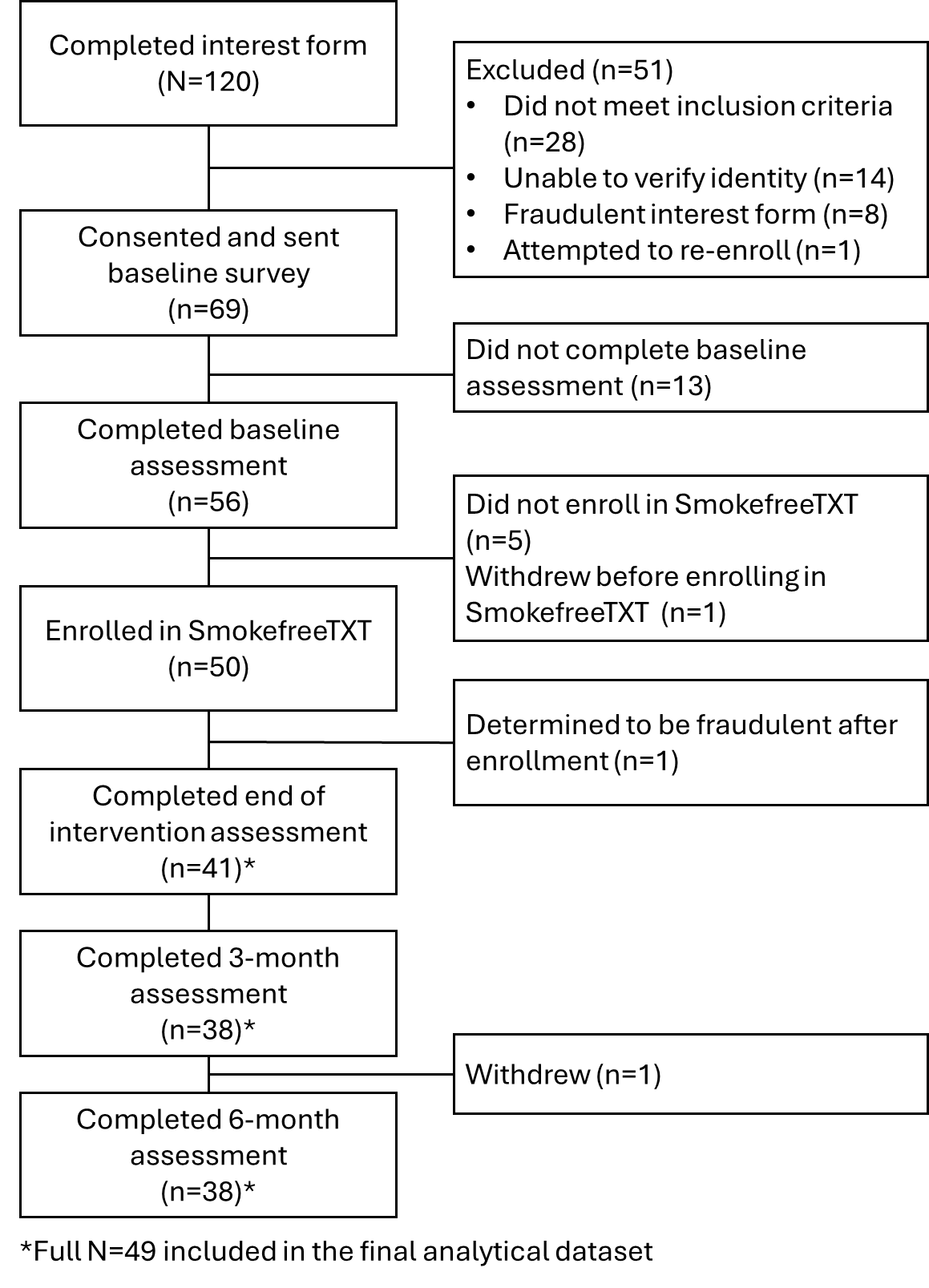


**Supplemental Figure 1. Study Flow diagram.** * Indicates that the full study sample (N=49) were included in the final analytic dataset for smoking cessation outcomes. Reason for exclusion provided at each stage where participants were removed from the study pool.
