# Supplementary material for: Pilot study examining the effect of rurality on engagement and abstinence for adult users of a text-message cessation intervention
Source: BMC Public Health. 2025 Nov 19;25:4061. doi: 10.1186/s12889-025-25284-6 (PMC12629046; doi:10.1186/s12889-025-25284-6)
Supplement: Supplementary file 2 — Supplementary Material 2. [file 12889_2025_25284_MOESM2_ESM.docx]

| **Supplemental Table 1: Logistic Regression Models of Cessation at All Survey Timepoints** | | | | |
| --- | --- | --- | --- | --- |
|  |  | End of Intervention | 3 Months | 6 Months |
|  |  | OR (95% CI) | OR (95% CI) | OR (95% CI) |
|  |  | n=49 | n=49 | n=49 |
| Rurality (Rural) |  | 4.13 (0.90,18.88) | 2.05 (0.45-9.39) | 3.16 (0.74-13.48) |
|  |  |  |  |  |
| Binge Drinking (Yes) |  | 4.87 (0.98,24.26) | 3.26 (0.66-15.73) | **5.49 (1.16-26.11)** |
|  |  |  |  |  |
| Time to First Cigarette (> 5 Minutes) |  | 2.60 (0.58, 11.61) | 1.79 (0.39-8.34) | 0.95 (0.24-3.85) |
|  |  |  |  |  |
| Confidence to Quit in Next 6 Months, Continuous |  | 1.57 (0.66,3.75) | 1.04 (0.45-2.39) | 1.59 (0.70-3.61) |
|  |  |  |  |  |
| Unmet Needs, Continuous |  | - | 3.21 (0.40-26.10) | - |
